# News and Events of the Week

**Published:** 1910-04-23

**Authors:** 


### News and Events of the Week.

King Albert has sent, says the Brussels correspondent of the *Times*, £500 to the Liverpool School of Tropical Medicine.

The annual meeting of the Zenana Bible and Medical Mission, or Indian Female Normal School and Instruction Society, will take place on Tuesday, April 26, at King's Chambers, Portugal Street, Kingsway, London, W.C.

Mr. John Reuben Lunn, M.R.C.S., medical superintendent of the St. Marylebone Infirmary, and Mr. Andrew Melville Paterson, M.R.C.S., Professor of Anatomy at the University of Liverpool, have been elected Fellows of the Royal College of Surgeons.

The second International Conference on Cancer Research will meet at Paris from October 1 to 5, and the fifth International Congress of Medical Electrology and Radiology will be held at Barcelona from September 13 to 18, under the patronage of King Alfonso.

The honorary gold medal of the Royal College of Surgeons has been awarded to Mr. Robert Fletcher in recognition of his distinguished labours in connection with the publication of the Index Medicus and the Index Catalogue of the Library of the Surgeon-General's Office, Washington, U.S.A. Mr. Fletcher became a member of the College in 1844.

The late Dr. Augustin Le Rossignol, M.D., C.M., L.R.C.P., M.R.C.S., of Jersey, formerly of the London Hospital, and Inspector-General of the Medical Staff of the Royal Jersey Militia, who died on February 18, at the age of 67, left personal estate in the United Kingdom valued at £11,872. Dr. Augustin Le Rossignol has left £100 to the Jersey Medical Society (Incorporated) upon trust.

A post-graduate course in school hygiene to meet the needs of those medical men and women who wish to qualify themselves for work under local education authorities has been arranged under the direction of Professor H. R. Kenwood, and began on Tuesday, April 19, at 3 p.m., at University College. In addition to the lectures there will be demonstrations and excursions. Visits will be paid to the Royal Sanitary Institute, where a special exhibition will be arranged, to some of the newer and special schools, and to the new buildings of University College School, Hampstead.

Sir Frederick Treves, who was the principal speaker at a meeting in Aldershot last week to establish an Aldershot and Farnborough First Aid Detachment of the British Red Cross Society, said that he was asking his hearers simply to make up their minds as to what they would do in the event of war, and not to leave the work of organisation until war actually broke out. Germany, France, Italy, and Russia all had Red Cross Societies. In England no organisation of the kind existed, and the reason alleged was that England would never be invaded. Quoting Sir Alfred Keogh, he said that the equipment for the training of a detachment should not cost more than £5. Lord Winchester's proposal that a voluntary aid detachment should be formed in Aldershot and Farnborough was adopted unanimously.

The China Emergency Appeal Committee, of which Sir Robert Hart is the president, reported that during March £2,081 had been received and promised towards the sum of £100,000 which the Committee is endeavouring to obtain for the development of medical and other training colleges for Chinese students. Among the contributions have been £770 from Edinburgh and £1,083 from Glasgow. The Committee have made the following grants :—For the Union Medical College, Peking, £2,000; the Union Medical College, Han-kau, £1,000; the Union Medical College, Mukden, £500; and the China Medical Missionary Association, £300.

The Council of the Royal College of Surgeons of England has awarded the Jacksonian Prize for the year 1909 to Mr. W. Girling Ball, F.R.C.S., of St. Bartholomew's Hospital, for his dissertation on "The Treatment of Surgical Affections by Vaccines and Antitoxins." The subject for the Jacksonian Prize for the year 1911 will be diseases of the pancreas, with special reference to their surgical treatment. The subject for the next Triennial Prize, which this year was not awarded, will be the anatomy and physiology of the pituitary body, and the relation with disease of its abnormal and morbid conditions.

A meeting on behalf of the Boys' Country Work Scheme will be held at Grosvenor House on Monday, April 25, at three o'clock. Mr. R. H. Yerburgh, M.P., will be in the chair, supported, among others, by the Archbishop of Westminster and the Duke of Norfolk. The Boys' Country Work Scheme was formed in 1908, its aim being to find good places in the country and the right boys to put in them. Both have been forthcoming, and to such an extent that the present appeal for funds is thoroughly justified. The excellent results produced by a small number of enthusiastic and devoted workers is in itself an eloquent demand for greater support so as to extend the scope of its usefulness.

At Leeds last week, before the Stipendiary Magistrate, a dispenser was charged with forging a death certificate. Mr. Arthur Willey, who prosecuted, said the prisoner was formerly with the late Dr. O'Neill in Burmantofts, and became associated with a large number of Dr. O'Neill's patients. He took a house, where he had the name "Forman" exhibited. He did not, however, describe himself as a medical man. Subsequently he got into touch with a medical practitioner, and it was arranged between them that they should divide equally the profits of the practice. Death certificates had been given by the prisoner in two cases in which the medical practitioner had not seen the patients at all, and bundles of certificates had been signed by the prisoner in respect of the health of school-children. A woman stated that on February 15 she went to a house in East Beckett Street, where the name "Forman" was over the door. She saw the prisoner, who looked at her child and said, "I won't make anything out. I don't think it is any use bringing it again, but if it is alive on Thursday bring it then." The child died the following day, and she went to ask for a certificate. The prisoner gave her a certificate, telling her to take it to the registrar, adding, "If the registrar says anything, don't tell him. Say I attended the child at its own home." Two other witnesses gave somewhat similar evidence. The accused, who pleaded "Not Guilty," was committed for trial at the Assizes.

Four cases of leprosy have been discovered recently among the aborigines of the Roeburne district in Western Australia. Two fatal cases occurred in the same place last year, but there is no quarantine or other station where the sufferers can live.

The current issue of the *St. Bartholomew's Hospital Gazette* contains an excellent and highly interesting contribution by Mr. Henry Rundle, F.R.C.S., on "Working under the Red Cross in the Franco-German War, 1870-71. Some Recollections." Mr. Rundle's description of Strasburg after the siege is particularly well done.

Dr. Tidswell, head of the Bureau of Microbiology at Sydney, has decided in the negative the accusation that the telephone mouthpiece is a harbour for germs. Dr. Tidswell examined fifty mouthpieces and obtained generally only negative results. Fifty animals were inoculated and of these thirteen died, one from hæmorrhage, and nine guinea-pigs from pneumonia.

Those who delight in long words will be interested in some of the recent additions to the new German Pharmacopœia. Aminobenzolydiaethylaminoaethanolum hydrochloricum is a drug extensively used nowadays, but most of us prefer to call it novocain, while any practitioner knows what pyramidon is, although we are not so sure if he would recognise it under the title of Pyrazolonumdimethylaminophenyldimethylicum, or eucaine under that of Trimethylbenzoxypiperidinum hydrochloricum.

The annual report of the Dean of the Faculty of Medicine to the Melbourne University Council shows that the number of students taking full courses in the medical school this year (1909) is the highest ever enrolled, being 326, of whom 15 are women. More and better clinical teaching being required, the University has taken the necessary steps to have the other general hospitals, the Alfred and the St. Vincent, recognised as clinical schools. Both hospitals have at once consented to begin the necessary buildings and improvements. The plans of the new Melbourne Hospital promise to give full facilities for teaching.

